# Geographic body size variation in ectotherms: effects of seasonality on an anuran from the southern temperate forest

**DOI:** 10.1186/s12983-015-0132-y

**Published:** 2015-12-23

**Authors:** Andrés Valenzuela-Sánchez, Andrew A. Cunningham, Claudio Soto-Azat

**Affiliations:** Centro de Investigación para la Sustentabilidad, Facultad de Ecología y Recursos Naturales, Universidad Andres Bello, República 440, Santiago, Chile; ONG Ranita de Darwin, Nataniel Cox 152, Santiago, Chile; Institute of Zoology, Zoological Society of London, London, NW1 4RY United Kingdom

**Keywords:** Hibernation hypothesis, Metabolic depression, *Rhinoderma darwinii*, Scaled mass index, Starvation resistance

## Abstract

**Background:**

Body size variation has played a central role in biogeographical research, however, most studies have aimed to describe trends rather than search for underlying mechanisms. In order to provide a more comprehensive understanding of the causes of intra-specific body size variation in ectotherms, we evaluated eight hypotheses proposed in the literature to account for geographical body size variation using the Darwin’s frog (*Rhinoderma darwinii*), an anuran species widely distributed in the temperate forests of South America. Each of the evaluated hypotheses predicted a specific relationship between body size and environmental variables. The level of support for each of these hypotheses was assessed using an information-theoretic approach and based on data from 1015 adult frogs obtained from 14 sites across the entire distributional range of the species.

**Results:**

There was strong evidence favouring a single model comprising temperature seasonality as the predictor variable. Larger body sizes were found in areas of greater seasonality, giving support to the “starvation resistance” hypothesis. Considering the known role of temperature on ectothermic metabolism, however, we formulated a new, non-exclusive hypothesis, termed “hibernation hypothesis”: greater seasonality is expected to drive larger body size, since metabolic rate is reduced further and longer during colder, longer winters, leading to decreased energy depletion during hibernation, improved survival and increased longevity (and hence growth). Supporting this, a higher post-hibernation body condition in animals from areas of greater seasonality was found.

**Conclusions:**

Despite largely recognized effects of temperature on metabolic rate in ectotherms, its importance in determining body size in a gradient of seasonality has been largely overlooked so far. Based on our results, we present and discuss an alternative mechanism, the “hibernation hypothesis”, underlying geographical body size variation, which can be helpful to improve our understanding of biogeographical patterns in ectotherms.

## Background

The pattern in which larger species within a genus are associated with colder climates forms part of a fundamental rule in biogeography: the Bergmann’s rule [1]. Although the mechanism originally proposed to explain this rule applied exclusively to endotherms, i.e. a larger body size is adaptive to colder climates because larger individuals need to produce less heat to maintain core body temperature over environmental temperature (“heat conservation” [1, 2]), the presence of the Bergmann’s cline has been evaluated (intra- and interspecifically) for both endothermic and ectothermic organisms [2–6]. Thereby, a trend of increased body size with increased latitude, which in most cases is associated with colder climates, has been found for many groups of birds and mammals [3]; whereas for ectotherms, a Bergmann’s cline [4, 7–9], a converse Bergmann’s cline [7, 10, 11], and absence of a cline [4, 12], have been described.

Despite the large amount of research related to Bergmann’s rule, most studies have aimed to describe trends rather than search for plausible explanations behind the observations [1]. The lack of a general pattern in ectotherms shows that it is unlikely that a single mechanism for geographic variation in body size exists for this group [9]. In this study we evaluated eight hypotheses currently proposed in the literature to account for intra-specific body size variation of ectotherms. We did this using body size data obtained from across the entire distributional range of an anuran species, the Darwin’s frog (*Rhinoderma darwinii* Duméril and Bibron 1841). These hypotheses were: (1) Starvation resistance: larger body sizes are adaptive to more seasonal environments where individuals spend a long time in inactivity (e.g. hibernating) because larger individuals have higher resistance to starvation (i.e. energy stores increase with size faster than metabolic rate) [7, 12–14]. (2) Heat balance: this hypothesis predicts that larger individuals would be favoured in cold environments due to their reduced surface/mass ratio and enhanced thermal inertia (with the exception of thermoconformers and larger ectothermic species) [9, 15, 16]. (3) Temperature-size rule: this hypothesis predicts that larger body sizes are associated with colder climates, since a negative relationship between ontogenetic temperature and size at maturity has been found in many ectotherms [17]. (4) Optimal body temperature: assuming a constant optimal body temperature in all individuals of a given species and that thermoregulation is a critical factor in cold but not in warm environments (hypothesis formulated for squamate reptiles), this hypothesis predicts smaller body sizes to be associated with colder environments, as the increased surface/mass ratio of smaller individuals permit more rapid heating and cooling, improving thermoregulatory capacity in cooler climates. Conversely, relaxing selective pressure on surface/mass ratio in warm environments permits individuals attains larger sizes, increasing other size-related benefits [5]. (5) Growing season length hypothesis: larger body sizes are expected in less seasonal environments where conditions and resources allow a longer period of growth [10]. (6) Primary productivity: this hypothesis predicts that areas with higher primary productivity are associated with an elevated food supply and therefore larger body sizes are reached [9, 16]. (7) Water availability: larger body sizes in amphibians are adaptive to drier environments because a lower surface/mass ratio reduces the loss of water [18, 19]. (8) Converse water availability: larger body sizes in amphibians are associated with wetter climates because activity in this group is associated with high water availability [9].

*Rhinoderma darwinii* is a small terrestrial amphibian that inhabits the temperate forests of Chile and Argentina [20]. Their populations are not homogeneously distributed, but clustered in specific areas; the species exhibits extremely high site fidelity [21]. *Rhinoderma darwinii* has developed a unique form of parental care, known as neomelia [21]; males incubate their offspring in their vocal sacs for the entire larval development, until metamorphosis takes place and juveniles are released to the environment [22]. Although this species had undergone rapid population declines in recent years, remnant populations continue to be distributed throughout much of its original range [20]. Marked geographical variations in body size have been observed, with adult snout-vent length ranging from 18.5 to 37.5 mm [C. Soto-Azat, personal communication], but this has not yet been formally evaluated.

In this study, we used morphometric and body condition data from >1000 adult *R. darwinii* captured across their entire distribution in Chile to demonstrate geographical body size variation. We then used an information-theoretic (I–T) modelling approach to evaluate competing hypotheses (described above) which might be driving this variation. The use of I–T methods is increasingly recommended in order to perform statistical inference using data from observational studies [23, 24]. Our results improve our understanding of the causes of intra-specific body size variation in *R. darwinii* and other ectothermic organisms.

## Methods

**Fig. 1:**
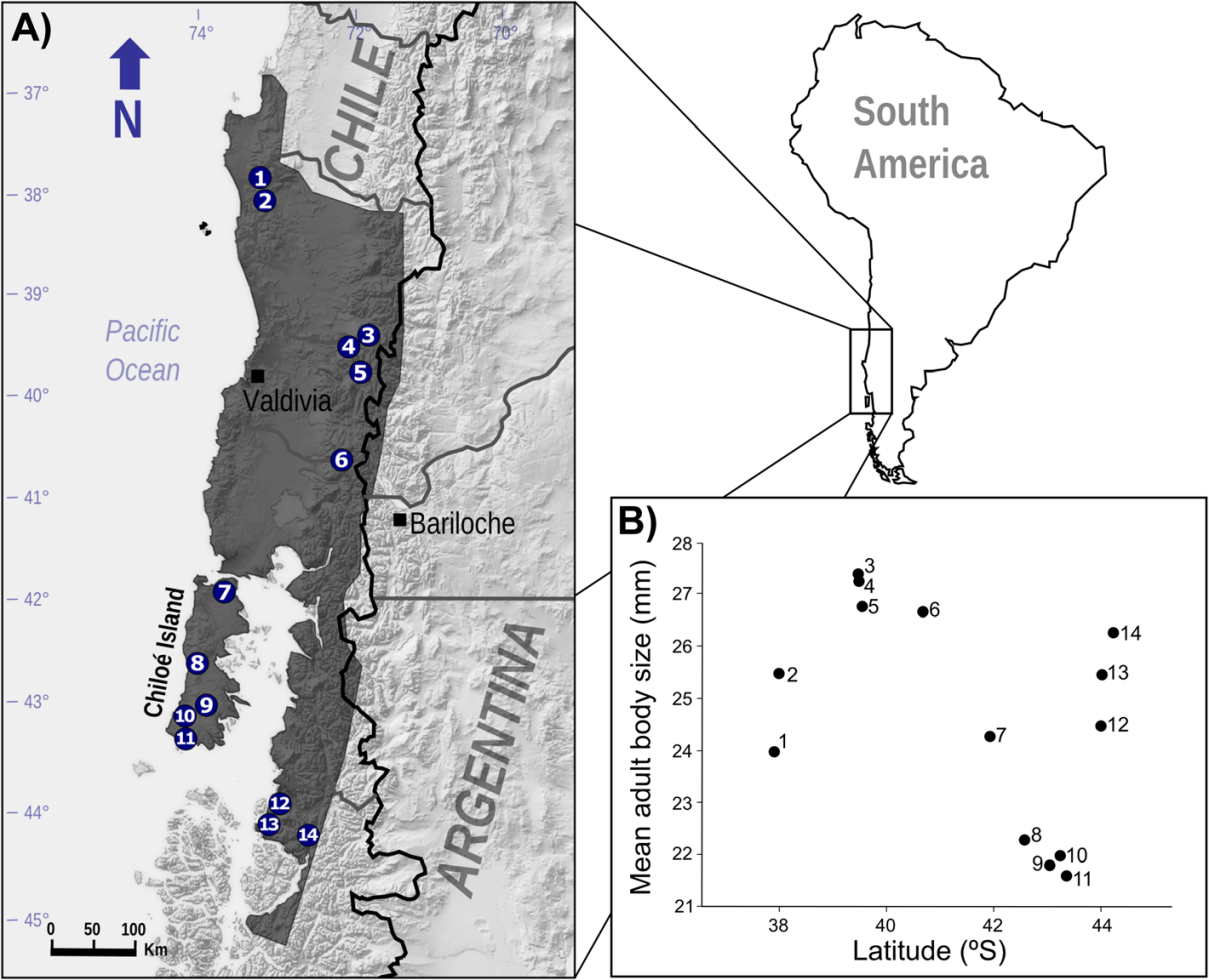
**a** Geographic distribution and (**b**) latitudinal body size variation (snout-vent length) of sampled *Rhinoderma darwinii* populations. The species distribution range is showed in the shaded area. (1) Natre (*n* = 13). (2) Contulmo (39). (3) Villarrica National Park (50). (4) Vergara hot springs (49; from Bourke, 2012). (5) Huilo Huilo (271). (6) Puyehue National Park (29). (7) Senda Darwin Biological Station (11). (8) Chiloé National Park (10). (9) Northern Tantauco (18). (10) Central Tantauco (29). (11) Southern Tantauco (177). (12) Northern Melimoyu (18). (13) Southern Melimoyu (170; from Crump, 2002). (14) Queulat National Park (127)

Twelve sites with populations of *R. darwinii* were surveyed between 2010 and 2014. Additionally, data from two sites: Southern Melimoyu (1998–1999) and Vergara hot springs (2007), were obtained from [25] and [26], respectively (Fig. 1a). All body size data were obtained between November and March, coincident with the reproductive season of the species [21]. The complete dataset comprised 1015 frogs from 14 sites covering most of the species’ range (Fig. 1a). The altitude of the sites ranged from 9 to 1120 m.a.s.l.

### Body size

All frogs were captured by hand and their snout-vent length (SVL) measured to the nearest 0.1 mm (0.5 mm for Crump’s data). Only SVL data was available from [25] and [26], whereas we obtained body mass (to the nearest 0.1 g) using a digital scale from the other 12 studied populations (this measure was used to calculate body condition, see below). Therefore, adult SVL was used as a proxy for adult body size. Adult stage was determined in each population according to morphologic (presence of vocal sac in males, enlargement of the coelomic cavity in gravid females) and behavioural characteristics (calling activity [21]). Because our SVL measurements were normally distributed in most sites, we used the mean as a measure of central tendency.

### Body condition

We used the scaled mass index (SMI [27]), a body condition index, as an indicator of the energy reserves present in each individual (i.e. fat and protein [27, 28]). The SMI accounts for the allometric relationship between body mass and body length, and has proved to be a good estimate of the true energetic condition [27–29]. Brooding males were excluded from all SMI analyses since their body mass is increased by the mass of incubating tadpoles. Therefore, the SMI data were calculated using body mass and SVL from 566 adults out of 12 sites. The regression coefficient (*b*SMA) was set adding juvenile body mass data to the complete dataset (total *n* = 725) because a better estimate of this parameter is obtained if the complete range of SVL variation is sampled [30]. Furthermore, SVL was standardized to mean adult SVL (23.7 mm).

### Environmental variables

The geographic coordinates of each site were obtained using a GPS receiver (eTrex Legend H, Garmin International Ltd., USA) or were identified from [25] and [26]. We used these coordinates to obtain climatic variables from a dataset built by [31]. These climatic surfaces were constructed with monthly temperature and rainfall data from ~1950 to 2000 and are available with a spatial resolution of 1 km^2^. As shown in Table 1, to construct our models we selected four climatic variables that are in association with the evaluated hypotheses: 1) annual mean temperature (BIO1), 2) temperature seasonality (BIO4), and 3) annual precipitation (BIO12). Additionally, we obtained the 4) Normalized Difference Vegetation Index (NDVI) of each site using images for 2010 (satellite Terra, MODIS sensor) with a spatial resolution of 30 seconds. The NDVI provides values that are highly correlated with photosynthetic mass and primary productivity [32]. We obtained images of the two mid-weeks of each month and the mean annual NDVI was subsequently calculated.

**Table 1:** Hypotheses for biogeographic variation in body size in ectothermic organisms

Hypothesis	Environmental variable	Predicted effect on body size
1. Heat balance	Annual mean temperature (BIO1)	Negative
2. Temperature-size rule	Annual mean temperature (BIO1)	Negative
3. Optimal body temperature	Annual mean temperature (BIO1)	Positive
4. Starvation resistance	Temperature seasonality (BIO4)	Positive
5. Growing season length	Temperature seasonality (BIO4)	Negative
6. Water availability	Annual precipitation (BIO12)	Negative
7. Reverse water availability	Annual precipitation (BIO12)	Positive
8. Primary productivity	NDVI^a^	Positive

Environmental variables used in this study to evaluate these hypotheses and their predicted effects on body size are presented

^a^Mean annual Normalized Difference Vegetation Index

### Data analyses

Data were first analysed to select models with a good fit to our data. Hence, we selected linear regression models because others (e.g. quadratic function) showed no improvement in terms of decrease residual variance (σ^2^).

Using the mean adult body size per site as dependent variable and bioclimatic and NDVI data as independent variables, we analysed a set of 11 candidate models for each of the eight hypotheses and all possible interactions (body size models, Table 2). As all the environmental variables we used are likely to impact the body condition of ectotherms, we used mean adult SMI per site as dependent variable and the bioclimatic and NDVI data as predictors to construct a set of body condition models (Table 3).

**Table 2:** Set of candidate linear regression models for mean adult body size (snout-vent length) in *Rhinoderma darwinii*

Model		adjusted *R* ^2^	*K*	AIC_c_	Δ_*i*_	*w*_*i*_
1	BIO4(+)	0.876	3	40.754	0.000	0.974
2	BIO1(−), BIO4(+)	0.872	4	49.005	8.252	0.016
3	BIO4(+), BIO12(+)	0.865	4	49.798	9.044	0.011
4	BIO1(−), BIO4(+), BIO12(−)	0.865	5	60.490	19.736	0.000
5	BIO1(−)	0.048	3	69.296	28.542	0.000
6	BIO12(+)	0.031	3	69.540	28.787	0.000
7	NDVI(+)	0.000	3	70.392	29.638	0.000
8	BIO12(+), NDVI(+)	0.042	4	77.219	36.466	0.000
9	BIO1(−), NDVI(+)	0.010	4	77.677	36.924	0.000
10	BIO1(−), BIO12(+)	0.000	4	78.108	37.354	0.000
11	BIO1(−), BIO12(+), NDVI(+)	0.000	5	89.098	48.345	0.000

The models are ranked by the AIC_c_ values from the best to the worst model. Predictor variable names (with the regression coefficient sign), adjusted *R* ^2^, number of estimated parameters (*K*), AIC_c_ values, AIC_c_ differences (Δ_*i*_) and Akaike weights (*w*_*i*_), are presented. Environmental variables included in models were: annual mean temperature (BIO1), temperature seasonality (BIO4), annual precipitation (BIO12), and the “Normalized Difference Vegetation Index” (NDVI)

**Table 3:** Set of candidate linear regression models for adult body condition (scaled mass index) in *Rhinoderma darwinii*

Model		adjusted *R* ^2^	*K*	AIC_c_	Δ_*i*_	*w*_*i*_
1	BIO4(+)	0.463	3	−7.950	0.000	0.552
2	BIO12(+)	0.401	3	−6.753	1.196	0.303
3	BIO4(+), BIO12(+)	0.673	4	−4.458	3.492	0.096
4	BIO1(−)	0.019	3	−1.331	6.619	0.020
5	NDVI(+)	0.000	3	0.020	7.970	0.010

Models with little support (*w*_*i*_ < 0.01) are not shown. The models are ranked by the AIC_c_ values from the best to the worst. Predictor variable names (with the regression coefficient sign), adjusted *R* ^2^, number of estimated parameters (*K*), AIC_c_ values, AIC_c_ differences (Δ_*i*_) and Akaike weights (*w*_*i*_), are presented. Environmental variables included in models were: annual mean temperature (BIO1), temperature seasonality (BIO4), annual precipitation (BIO12), and the “Normalized Difference Vegetation Index” (NDVI)

We compared our competing models (using both body size and body condition sets) through the Akaike’s Information Criterion corrected for small-sample size (AICc [33]), which is an estimator of the relative expected Kullback-Leibler distance [23]. We calculated the AICc difference as: ${\varDelta}_i = \mathsf{AI}{\mathsf{C}}_i\hbox{--}\ \mathsf{AI}{\mathsf{C}}_{min}$. To interpret the relative likelihood of a model we used Akaike weights (*w*_*i*_), which are a normalization of the likelihood of a model given the data [23]. To compare the relative likelihood of one model vs. another, we used the evidence ratio (i.e. the evidence ratio for model *a* vs. model *b* is *w*_*a*_/*w*_*b*_ [24]).

In order to provide a simple interpretation of the independent effect on adult body size of each predictor variable, we performed a hierarchical partitioning analysis (HP [34]). Moreover, HP alleviates multicollinearity between predictor variables [35]. This analysis estimates the independent contribution of each predictor variable by performing all possible combinations of linear models of *x*_*s*_ and *y* [36]. We used R (see below) to perform the HP using the public domain ‘hier.part package’ (v 1.0-4 [36]).

Spatial autocorrelation in body size, body condition models and residuals were assessed using the Moran’s *I* coefficient with a Monte Carlo permutation test (199 permutations) for significance evaluation. Additionally, spatial correlograms were constructed using 10 distance classes [37].

All statistical analyses were performed using R (v 2.11.1) or SAM (v 4.0 [37]).

## Results

### Body size

**Fig. 2:**
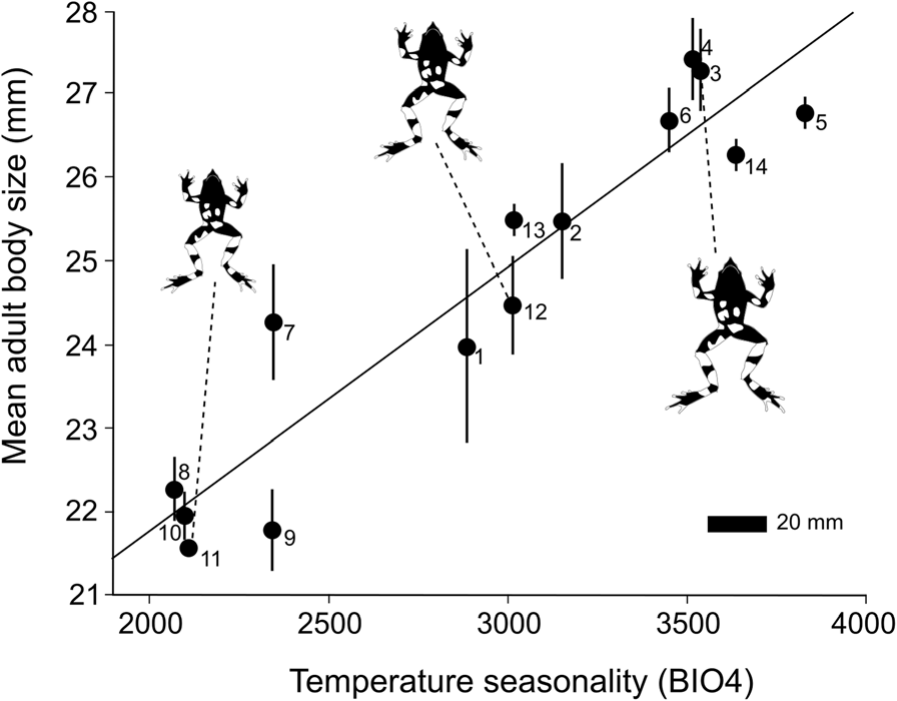
Relationship between temperature seasonality and adult body size (snout-vent length; mean ± SE) in 14 *Rhinoderma darwinii* populations. Drawings represent the frog body size in a ventral view. (1) Natre. (2) Contulmo. (3) Villarrica National Park. (4) Vergara hot springs. (5) Huilo Huilo. (6) Puyehue National Park. (7) Senda Darwin Biological Station. (8) Chiloé National Park. (9) Northern Tantauco. (10) Central Tantauco. (11) Southern Tantauco. (12) Northern Melimoyu. (13) Southern Melimoyu. (14) Queulat National Park

**Fig. 3:**
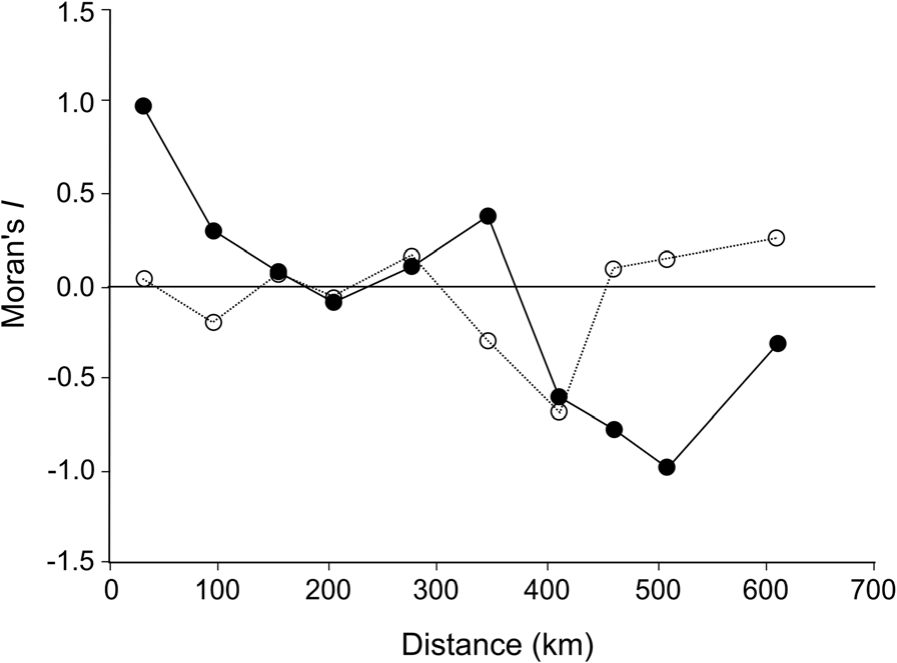
Spatial correlograms using Moran’s *I* for mean adult body size (Snout-to-vent length; *solid circles*) and residuals (*open circles*) of the best ranked model for body size variation in *Rhinoderma darwinii*

We captured a total of 796 different adult frogs between 2009 and 2014. Additionally, the mean body size of 170 and 49 adults from Southern Melimoyu and Vergara hot springs, respectively, were included in our dataset (totalling 1015 individuals). Sample size per site ranged from 10 to 271. A clear geographic variation in body size was observed, with mean population adult body size ranging from 21.6 mm to 27.4 mm (Fig. 2). Adult body size was spatially autocorrelated only in the smaller distance class (Fig. 3, Monte Carlo permutation test, *P* = 0.005), but spatial autocorrelation did not show an expected latitudinal cline because Moran’s *I* values did not decrease linearly with increased distance (Fig. 3). Thus, smaller mean body sizes were found in latitudinal intermediate areas, such as Chiloé Island (Fig. 1b).

**Fig. 4:**
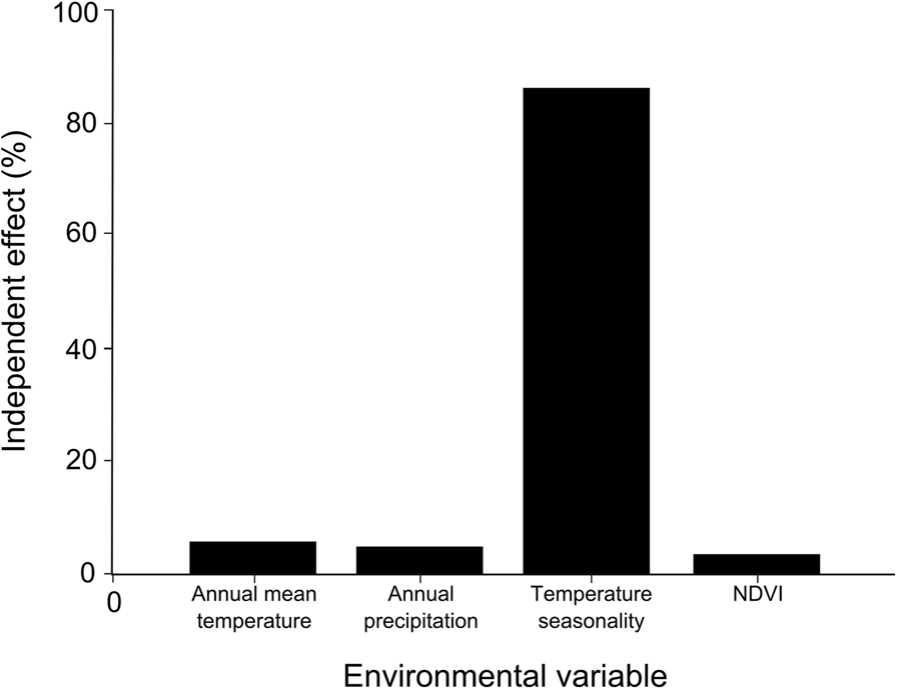
Independent effects (in percentage) of four environmental variables over adult mean body size of *Rhinoderma darwinii* calculated with hierarchical partitioning

Given the set of candidate models and based on evidence ratios, the best model for body size variation was 61.9 and 92 times more likely than the second and the third best ranked models, respectively and 19,306 and 1,576,984 times more likely than the fourth and the fifth next models. According to our analyses, the best model for our dataset included only temperature seasonality as a predictor variable. This variable was also incorporated (but not alone) in the next three best models (Table 2). A positive relationship between temperature seasonality and adult body size was found in all models that included this variable. Consistent with the results of the I-T approach, the independent contribution of temperature seasonality was clearly higher than any of the other predictor variables (Fig. 4). The second best model incorporated additionally the annual mean temperature, which was negatively related to adult body size. The third best model included a positive relationship between annual precipitation and adult body size. The best ranked model that incorporated NDVI was 2,727,947 times less likely that the best model. The residuals of the best model were not spatially autocorrelated (Fig. 3, Monte Carlo permutation test, *P* = 0.834), therefore no bias in the computation of the AIC_c_ values were expected [9, 15].

### Body condition

Body mass scaled allometrically with SVL with a *b*SMA of 3.278. The adult SMI was not correlated with body size (*r* = 0.020, *n* = 566, *P* = 0.642). The mean adult SMI per site ranged from 0.77 to 1.33. Only five linear regression models had a *w*_*i*_ higher than 0.01 (Table 3). The most parsimonious model to explain body condition variation was just 1.8 and 5.7 times more likely than the second and third subsequent ranked models. These three models included temperature seasonality, annual precipitation, and a combination of both, as predictor variables (Table 3). The sum of the *w*_*i*_ of all models that included temperature seasonality and annual precipitation were 0.661 and 0.405, respectively; whilst these values were only 0.027 and 0.022 for mean annual temperature and NDVI, respectively. No spatial autocorrelation was found for adult SMI or the residuals of the best model (Monte Carlo permutation test, *P* = 0.111 and *P* = 0.317, respectively).

## Discussion

We identified clear evidence of geographic variation in body size of *R. darwinii* across its range and we found strong evidence for a single model, comprising only temperature seasonality as a predictor variable (Fig. 2) to account for this (i.e. *w*_*i*_ > 0.9 [23]). Only two additional models provided support (both *w*_*i*_ <0.02), whereas the remaining eight models tested gave no support (the sum of all *w*_*i*_ was <0.001). Temperature seasonality was present in all three supportive models and this variable had a high independent effect (86.1 %) indicating that it is likely the most important factor determining the body size variation pattern observed. Interestingly, the residuals of the best model were not spatially autocorrelated suggesting the absence of an additional and independent, spatially patterned variable as an important factor determining body size variation in this species.

The observed positive relationship of temperature seasonality and adult size in *R. darwinii* (Fig. 2) is in accordance with the starvation resistance hypothesis (hereafter SRH), which predicts that, because energy reserves increase faster than energy depletion as body size increases, larger individuals are expected in more seasonal environments where animals experience long periods without food (e.g. during aestivation or hibernation [7, 12–14]); i.e. energy storage is iso- or hyperallometric (*b* ≥ 1) whereas metabolic rate is hypoallometric (*b* < 1). The evidence supporting this hypothesis in ectotherms is scarce, although some experimental studies show that this is true at least for some species of flies and antlions [14, 38, 39]. In contrast, and associated with their low foraging efficiencies, larger individuals of the Galapagos marine iguana (*Amblyrhynchus cristatus* Bell 1825) experience higher mortality rates compared with medium-sized individuals when food is scarce [40]. Furthermore, [41] showed that change in metabolic rate over time in the marine fish *Menidia menidia* (Linnaeus 1766) is unlikely to be determined by a single allometric function. Energy depletion in *M. menidia* at 4 and 8 °C was initially greater in small compared to large individuals; however, as time progressed (fishes followed up to 127 days) this pattern reversed and by the end of the experiment, differences in energy depletion had disappeared [41].

One problem of applying SRH over a broad biogeographical scale is that both seasonality and metabolic rate are commonly correlated with temperature [42–44]. For instance, in our study area temperature seasonality was negatively correlated with the mean temperature of the coldest quarter (Pearson’s *r* = −0.650, *P* = 0.012, *n* = 14). Using data from [45] to calculate standard metabolic rate in amphibians (i.e. Q_10_ = 2.21, standard metabolic rate at 20 °C = 0.081 mL O_2_ h^−1^, allometric exponent *b* = 0.884) allows us to illustrate this inconsistency: during the winter months an individual of *R. darwinii* from the area with lowest seasonality (Southern Tantauco; mean temperature of coldest quarter = 7.6 °C) weighing 1.0 g would have a metabolic rate 1.43 higher than a frog from the area with greatest seasonality (Villarrica National Park, mean temperature of coldest quarter = 2.9 °C) having the same weight. This metabolic rate increase in the least seasonal environment can decrease post-hibernation body condition of animals, thus driving selection for larger bodies according to the SRH, unless: 1) energy storage before, or energy intake during, the winter months exceeds energy depletion, or 2) there are genetic differences in metabolic rate between locations.

An alternative, non-exclusive hypothesis regarding geographical differences in body size as a response to seasonality appears to be plausible in light of our data and previous evidence from ectotherms (mainly amphibians). This novel hypothesis, termed the “hibernation hypothesis” (HH) predicts the same pattern as the SRH, i.e. within an ectothermic species, larger body sizes are found in areas of greater seasonality and colder winters, but the mechanisms underlying these hypotheses are somewhat different. While the SRH assumes that body size variation is the result of an adaptive response to the stressful conditions of living in a seasonal environment, the HH assumes that the variation in body size is related to temperature-dependent differences in metabolic rate. Furthermore, the HH also predicts that post-overwinter body condition should be better in animals from areas with lower mean winter temperatures. The HH is applicable to ectotherms living in seasonal areas (e.g. temperate and polar zones), where food availability decreases dramatically during winter and where metabolism is supported only by energy storage. Many species in these areas overwinter in a depressed metabolic state (e.g. hibernation), which is a physiological adaptation for survival during extended periods of inactivity [44]. We do not have data about hibernation duration and it geographic variation in *R. darwinii*, but several pieces of evidence support the occurrence of hibernation in this species: 1) an ongoing study has found that frogs have a lower recapture probability in winter in comparison with summer months and this period of inactivity appears to be longer in more seasonal areas [A. Valenzuela-Sánchez, unpublished observations], 2) some populations live in areas which are covered by a thick layer of snow during the winter months (e.g. Huerquehue and Villarrica National Parks), and 3) Busse [46] provides observations of this condition in an outdoor captive facility maintained in Bonn, Germany. While some authors hypothesize that individuals that hibernate for longer periods need to invest most in energy storage and self-maintenance [47], the opposite is more likely to occur in ectotherms if the temperature effects on metabolism are considered [44, 47]; i.e. the rate of depletion of energy reserves and the probability of death by starvation are expected to be lower in more seasonal areas with colder winters [48–50]. This decreased mortality rate leads to greater longevity which permits growth over a longer period of time resulting in larger body size [51], but it is also important to note that growth rate can also be increased [52], probably because more energy is available to be allocated to growth. There is some empiric evidence supporting the HH. For instance, field observations over a 23-year period of the common toad (*Bufo bufo* Linnaeus 1758) in the United Kingdom, suggested a decreased depletion rate of energy reserves and decreased mortality rates during colder winters compared to milder winters [50]. Similarly, in common frogs (*Rana temporaria* Linnaeus 1758) in Sweden, post-hibernation energy stores are larger in higher latitudes where winters are colder and longer and where frogs consequently hibernate for longer periods [53]. In our study, body condition in *R. darwinii* was positively correlated with temperature seasonality, i.e. better mean body conditions were found in more seasonal areas.

As observed for other ectothermic species, energy storage can also reflect genetic based differences in the allocation and depletion of energy [41, 53]. It is reasonable, however, to expect that any such genetic signal would be detected as a spatially patterned variable leading to spatial autocorrelation in body condition, which was not the case in our study. Further investigations are required to directly assess this apparent lack of genetic based variation in the metabolism of *R. darwinii*. Such studies may contribute to elucidate this confounding factor, perhaps providing additional support to the HH.

Large body size can be advantageous during long stressful periods due to reasons additional to energy consumption, e.g. decreasing desiccation rates by body-mass-to-surface-area ratio effects [19]. For instance, it takes larger post-metamorphosed Couch’s spadefoot toads (*Scaphiopus couchii* Baird 1854) longer to reach a critical level of dehydration than smaller individuals [18]. The role of desiccation rates, and possibly other factors, during hibernation in shaping adult body size in ectothermic organisms is unknown, but should not be overlooked. Additionally, other factors related to seasonality could be influencing adult body size in ectotherms. For example, predation pressure could be maintained for longer periods in less seasonal environments, decreasing the probability to survive and reach larger body sizes in animals with indeterminate growth. Furthermore, the stress-related effects associated with predation risk can decrease body size by altering development and growth rates [54, 55].

## Conclusions

Our analyses showed that, of the hypotheses tested, the starvation hypothesis is the most consistent with our results of adult body size variation in *R. darwinii*. Considering the known role of temperature on ectothermic metabolism, however, we formulated a new, non-exclusive hypothesis, regarding intraspecific body size variation in ectothermic animals in temperate areas. The HH predicts a larger adult size and better post-hibernation body condition in individuals from more seasonal areas and with colder winters. The mechanisms underlying the HH are the temperature-dependence of metabolic rate and its reduction during hibernation [43, 44]. It is surprising that, despite the largely recognized effects of temperature on metabolic rate ([42, 43], but see [56]), its importance as a determinant of body size in a gradient of seasonality (and therefore of hibernation duration) has been ignored so far. We do not fully understand the relative importance of lower rates of energy reserve depletion in larger animals (SRH), or the improved survival and longevity (and hence growth) of animals exposed to longer, colder winters (HH) in determining body size variation in *R. darwinii* or other ectothermic species, but it is likely that the latter plays a relevant role. We, therefore, provide a plausible additional mechanism underlying geographic body size variation in ectothermic organisms, which can improve our understanding of body size biogeographical patterns and the mechanisms underlying these.
